# Cardiometabolic health during early adulthood and risk of miscarriage: a prospective study

**DOI:** 10.12688/wellcomeopenres.16245.2

**Published:** 2021-02-12

**Authors:** Maria C. Magnus, Diana D. S. Ferreira, Maria Carolina Borges, Kate Tilling, Deborah A. Lawlor, Abigail Fraser

**Affiliations:** 1 Centre for Fertility and Health, Norwegian Institute of Public Health, Oslo, 0213, Norway; 2 MRC Integrative Epidemiology Unit, University of Bristol, Bristol, UK; 3 Population Health Sciences, Bristol Medical School, University of Bristol, Bristol, UK; 4 NIHR Bristol Biomedical Research Centre, Bristol, UK

**Keywords:** blood pressure, body-mass index, cholesterol, fatty acids, miscarriage.

## Abstract

**Background:** Several studies have found that women who are overweight or obese have an increased risk of miscarriage. There is also some evidence of associations of other aspects of cardiometabolic health, including blood pressure and lipids, with miscarriage risk, although these have not been examined to the same extent as body-mass index (BMI).

**Methods:** Our objective was to investigate the risk of miscarriage according to pre-pregnancy cardiometabolic health. We examined pre-pregnancy levels of BMI, blood pressure, fasting insulin and metabolites profile at age 18 and risk of miscarriage by age 24. The study included adult female offspring in the Avon Longitudinal Study of Parents and Children with a pregnancy between 18 and 24 years of age (n=434 for BMI and blood pressure; n=265 for metabolites). We used log-binomial regression to calculate adjusted associations between cardiometabolic health measures and miscarriage.

**Results:** The overall risk of miscarriage was 22%.  The adjusted relative risks for miscarriage were 0.96 (95% CI: 0.92-1.00) for BMI (per unit increase), 0.98 (0.96-1.00) for systolic blood pressure, and 1.00 (0.97-1.04) for diastolic blood pressure (per 1 mmHg increase).  Total cholesterol, total lipids and phospholipids in HDL-cholesterol were associated with increased likelihood of miscarriage, but none of the p-values for the metabolites were below the corrected threshold for multiple testing (p-value ≤0.003).

**Conclusions:** Our findings indicate no strong evidence to support a relationship between pre-pregnancy cardiometabolic health and risk of miscarriage in young, healthy women who became pregnant before age 24. Future studies are necessary that are able to evaluate this question in samples with a wider age range.

## Introduction

An estimated 12–15% of recognized pregnancies end in miscarriage ^1, 2^. Despite extensive research efforts, we know very little about its aetiology. It is believed that genetic components, including parental chromosomal rearrangements and abnormal embryonic genotypes or karyotypes, explain about half of early miscarriages ^3^. Risk of miscarriage is nonlinear in relation to women’s age; the risk is slightly higher in young women (<20 years), with a nadir of risk in the mid-20s, before an approximately linear increase from age 30 ^4^.

Pre-pregnancy cardiometabolic health is likely to affect chances of conception as well as risk of fetal loss and may therefore be a key factor in ensuring a successful pregnancy ^5, 6^. Several studies have found that women who are overweight or obese have an increased risk of miscarriage, while others report a potential nonlinear relationship with an increased risk in underweight women as well ^7, 8^. There is conflicting evidence of a relationship between blood pressure and risk of miscarriage, with one preconception cohort indicating a positive association with pregnancy loss ^9^, while another study reported a lower blood pressure among women with a history of recurrent miscarriage ^10^. The evidence of a relationship between lipid levels and risk of miscarriage is scarce. Animal studies support the notion that treatment of high cholesterol with statins may reduce the risk of miscarriage ^11^.

The aim of the current study was to examine whether pre-pregnancy body-mass index (BMI), blood pressure, fasting insulin and metabolomic profile at age 18 are associated with miscarriage risk in young, healthy women who became pregnant before 24 years of age. The Avon Longitudinal Study of Parents and Children provided a unique opportunity to study this research question, since the adult offspring (also referred to as Generation 1, G1) of the women recruited to this pregnancy cohort in the 1990’s (G0) are now having their own children (G2), and their preconception cardiometabolic health has been extensively assessed.

## Methods

### Avon Longitudinal Study of Parents and Children

**Figure 1.**
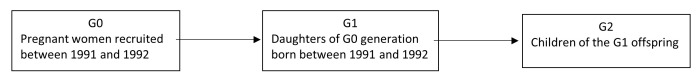
Illustration of the study design and the generations included in the Avon Longitudinal Study of Parents and Children.

We studied female offspring (G1) participating in the Avon Longitudinal Study of Parents and Children (ALSPAC) ^12^. Pregnant women (G0) resident in Avon, UK with expected delivery date between 1 ^st^ of April 1991 and 31 ^st^ of December 1992 were invited to participate. The initial participation rate was estimated to be 75%, and a total of 14,541 pregnancies were enrolled into the cohort. There was a total of 14,676 fetuses, resulting in 14,062 live births and 13,988 children who were alive at 1 year of age. Information from participating mothers (G0) and their offspring (G1) were collected through self-completed questionnaires at regular intervals. G1 offspring were also invited to participate in regular clinical examinations from age 7 onwards. After age 22, study data were collected and managed using REDCap electronic data capture tools hosted at the University of Bristol ^13^. The G1 generation have now reached an age where they are starting to have their own children. Figure 1 depicts the study design and generations in the ALSPAC cohort. The current study included 434 adult female offspring (G1) from the ALSPAC cohort who had a pregnancy by age 24 and from whom measures of cardiometabolic health at 18 years of age were available ( *Extended data*, Appendix 2) ^14^. Ethical approval for the data collection in ALSPAC was obtained from the ALSPAC Ethics and Law Committee and the Local Research Ethics Committee. Patients or the public were not involved in the design, or conduct, or reporting, or dissemination plans of our research.

### Measures of cardiometabolic health at 18 years of age

When G1 participants were an average of 18 years of age (SD 0.4 years), they were invited to a comprehensive clinical examination that included blood sampling. Trained study nurses measured the participant’s height and weight, from which we calculated their BMI (weight in kg/height in metres squared). Blood pressure (systolic and diastolic) was measured at least twice on the left arm. We used the mean systolic (SBP) and diastolic (DBP) blood pressure from the last two measurements taken with the DINAMAP 9301 machine as the exposures. 

Fasting (overnight or minimum of 6-hours) blood samples were immediately spun and frozen at -80°C. Comprehensive metabolomic profiling was conducted at the University of Glasgow using a high-throughput proton nuclear magnetic resonance platform on an NMR spectometer. Two different pulse sequences are used. A 1D nuclear overhauser effect spectroscopy pulse sequence is used to detect lipoproteins. A Carr Purcell Meiboom Gill sequence is used to detect low-molecular-weight metabolites. This platform measures a total of 154 metabolomic traits, representing a broad range of signatures of systemic metabolism and 14 lipoprotein subclasses (particle concentration, lipid concentrations and composition), fatty acids and fatty acid compositions, amino acids, ketone bodies, glycolysis and glucogenesis-related metabolomic traits. The various metabolites that are part of the panel has been described in detail elsewhere ^15^. We also included insulin as an exposure, which was measured separately from the metabolomics panel using an ELISA (Mercodia, Uppsala, Sweden) assay (catalogue number 10-1113-01) that does not cross react with proinsulin and plasma glucose. The read optical density was 450 nm.

Correlations between measures of cardiometabolic health at 18 years of age ( *Extended data*, Appendix 1) ^14^ were in line with what we expected based on the literature ^16, 17^.

### Identification of pregnancies and miscarriage by age 24

Information on whether participants had been pregnant, and the outcome of their pregnancies, was obtained by self-report at age 21, 22, 23, and 24. The outcome of any pregnancies were classified as “baby born alive”, “baby born stillborn”, “termination of an unwanted pregnancy”, “termination for medical reasons” and “miscarriage”. No additional information on the gestational week when the pregnancy ended or the birthweight of the offspring was available to better distinguish between stillbirth and miscarriage. The outcome of interest was any miscarriage reported between age 18 and 24. The reference group consisted of all other pregnancy outcomes (including induced abortions).

### Covariates

We identified potential confounders that could be associated with both cardiometabolic health and risk of miscarriage. This included age (continuous), smoking status (never, weekly, daily) and a marker of socioeconomic position at the time of the cardiometabolic assessment at age 18: whether the woman herself was “Not in Education, Employment or Training (NEET; yes versus no). Due to the young age of the women when their cardiometabolic health was assessed (age 18), they were mostly still in school, and information on their NEET status was therefore used as opposed to completed educational qualifications. However, we also considered the woman´s mother´s educational qualifications as a measure of family socio-economic position, categorized into low (CSE and vocational), medium (O or A level) and high (university degree).

### Statistical analysis

We examined the evidence for a nonlinear relationship by comparing the log-likelihoods of models including tertiles of the measures of cardiometabolic health versus a single linear term. We estimated associations using log-binomial regression, reporting the relative risks (RR) for miscarriage per unit increase in BMI, SBP, and DBP adjusting for age and maternal educational qualifications. SBP and DBP were further adjusted for BMI measured at the same time point. We conducted complete-case analysis, as the proportion of individuals with missing information was low for age and maternal educational level (<2%). All of the metabolites were internally standardized before analysis, and we report the RR for miscarriage per standard deviation increase in the metabolites, after adjusting for the same covariates mentioned above. The associations of blood pressure and metabolites with risk of miscarriage was also adjusted for the participant’s BMI at 18 years. We further conducted sensitivity analyses adjusting for the women’s smoking status and NEET at age 18 in a subsample for which these data were available (approximately 80%). We also conducted an analysis excluding all women with self-reported hypertension, high cholesterol or diabetes at the time of the cardiometabolic health assessment, to evaluate the potential influence of the subgroup of women who reached the threshold for these clinical definitions on the estimated associations. As there might be underlying genetic components influencing the risk of miscarriage, we also conducted a sensitivity analysis adjusting for maternal (G0) number of miscarriages. Due to the large number of tests conducted, we accounted for multiple testing. The metabolites in the metabolomics panel are strongly correlated. A principal components analysis found that the first 17 principal components explained around 95% of the variation in the metabolites, and this was therefore estimated to be the number of independent tests. The conventional p-value threshold of 0.05 was therefore divided by 17 to yield a corrected threshold of 0.003 ^18^. 

All analyses were conducted using Stata version 15 (Statacorp, Texas).

## Results

Of the women who attended the 18-year clinic assessment and who provided information on their obstetric history (n=2,243), 465 (21%) had experienced a pregnancy ( *Extended data*, Appendix 2) ^14^. The overall risk of miscarriage among women who had been pregnant was 22% (n=94 women). *Extended data*, Appendix 3 ^14^ presents characteristics for the following: all female G1 participants, female G1 participants who attended the 18 year clinic assessment with a known pregnancy history female offspring who had experienced a pregnancy between 18 and 24 years of age, and female G1 participants included in the final analyses. Women with information on any of the exposures of interest (n=434) had less educated mothers and a higher mean BMI, when compared to all female offspring in ALSPAC (n=6,838). They were also more likely to smoke when they were 18 years of age. The distribution of background characteristics according to whether the woman experience a miscarriage or not is shown in Table 1.

**Table 1.** Distribution of background characteristics according to risk of miscarriage among women who have had at least one pregnancy.

Background characteristics	No miscarriage (n=340)	At least one miscarriage (n=94)	p-value
**Age at 18 year follow-up**	17.8 (0.4)	17.8 (0.4)	0.463
**Maternal education**			0.021
Low	96 (28.2)	30 (31.9)	
Medium	221 (65.0)	50 (53.2)	
High	23 (6.8)	14 (14.9)	
**Smoking at age 18**			0.037
No	199 (58.5)	46 (48.9)	
Weekly	19 (5.6)	2 (2.1)	
Daily	68 (20.0)	31 (33.0)	
Missing	54 (15.9)	15 (16.0)	
**Maternal history of miscarriage**			0.258
0	259 (76.2)	68 (72.3)	
1	45 (13.2)	17 (18.1)	
2 or more	23 (6.8)	3 (3.2)	
Missing	13 (3.8)	6 (6.4)	
**Not in education, training or** **working at age 18**			0.360
No	259 (76.2)	69 (73.4)	
Yes	16 (4.7)	8 (8.5)	
Missing	65 (19.1)	17 (18.1)	
**BMI at age 18**	24.0 (4.8)	23.0 (4.8)	0.101
**DBP at age 18**	65.0 (6.4)	64.4 (6.1)	0.430
**SBP at age 18**	114.7 (8.9)	112.5 (8.7)	0.038

**Figure 2.**
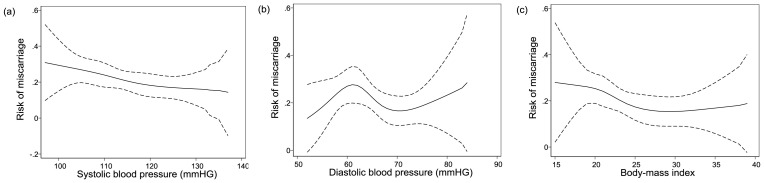
Risk of miscarriage by 24 years of age according to blood pressure and body-mass index at 18 years among adult female offspring in the Avon Longitudinal Study of Parents and Children born between 1991–1992.

The mean SBP was 114.2 mmHg (SD 8.9), the mean DBP was 64.9 mmHg (SD 6.3), while the mean BMI was 23.7 kg/m ^2^ (SD 4.9). The risk of miscarriage seemed to decrease in a linear fashion with increasing SBP ( Figure 2). For DBP, the smoothed plot of the risk of miscarriage showed some evidence of a nonlinear relationship before multivariable adjustment ( Figure 2), but there was no statistical evidence to support a nonlinear association when this was formally tested (p-value 0.4). The adjusted RRs for miscarriage was 0.98 (95% CI: 0.96-1.00) per mmHg higher SBP, and 1.00 (95% CI: 0.97-1.04) per mmHg higher DBP ( Table 2). There was no evidence of a difference in risk of miscarriage by BMI ( Figure 2), with an adjusted RR of 0.96 per kg/m ^2^ increase (95% CI: 0.92-1.00) ( Table 2).

**Table 2.** Association between body-mass index and blood pressure at age 18 with risk of miscarriage by age 24 among adult female offspring in the Avon Longitudinal Study of Parents and Children born between 1991–1992. The coefficient reflect the change in risk of miscarriage per unit increase in the exposure.

Exposure	N (n with miscarriage)	Mean (SD)	Unadjusted RR (95% CI)	Adjusted ^*^ RR (95% CI)	Adjusted ^†^ RR (95% CI)
**Body-mass index (kg/m ^2^)**	424 (89)	23.7 (4.9)	0.96 (0.92-1.01)	0.96 (0.92-1.00)	NA
**Systolic blood pressure** **(mmHg)**	418 (91)	114.2 (8.9)	0.98 (0.96-1.00)	0.97 (0.95-1.00)	0.98 (0.96-1.00)
**Diastolic blood pressure** **(mmHg)**	418 (91)	64.9 (6.3)	0.99 (0.96-1.02)	0.99 (0.96-1.02)	1.00 (0.97-1.04)

^*^ Adjusted for age and maternal education. ^†^ Adjusted for age, maternal education and BMI at 18 years.

**Figure 3.**
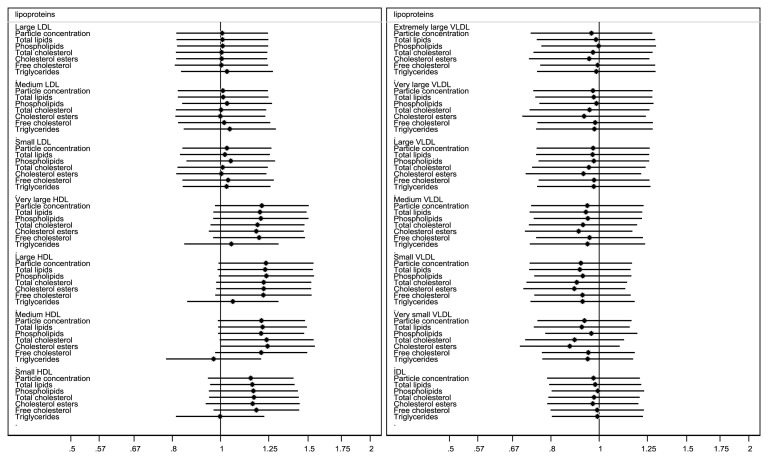
Relative risks for miscarriage according to components of lipoproteins among adult female offspring in the Avon Longitudinal Study of Parents and Children born between 1991–1992. The Relative risks for miscarriage reflect the risk per standard deviation increase in the exposure of interest. The estimates are adjusted for age, maternal educational qualifications, and BMI at 18 years. The analysis of metabolites included 265 women (among whom 63 experienced a miscarriage).

**Figure 4.**
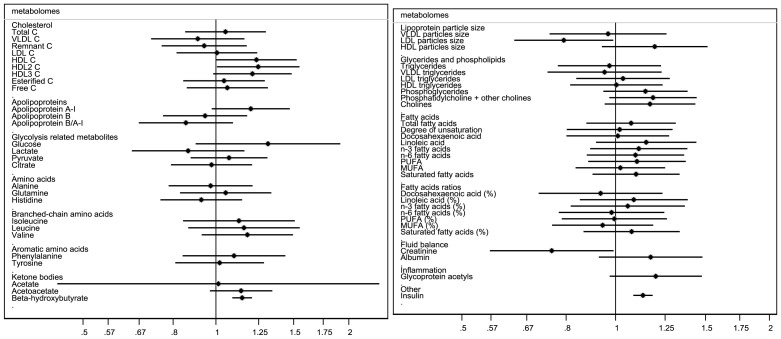
Relative risks for miscarriage according to other components of the metabolomics panel among adult female offspring in the Avon Longitudinal Study of Parents and Children born between 1991–1992. The Relative risks for miscarriage reflect the risk per standard deviation increase in the exposure of interest. The estimates are adjusted for age, maternal educational qualifications, and BMI at 18 years. The analysis of metabolites included 265 women (among whom 63 experienced a miscarriage).

We examined a large number of metabolites in relation to the risk of miscarriage (information available for 154 women). Figure 3 shows the associations of components of high-density lipoprotein (HDL), low-density lipoprotein (LDL), intermediate-density lipoprotein (IDL) and very low-density lipoprotein (VLDL)-cholesterol with risk of miscarriage, while Figure 4 shows the associations of non-lipoprotein metabolic measures with miscarriage. A total of nine of the metabolites were associated with the risk of miscarriage at the nominal level (p-value <0.05). This included concentrations of medium HDL cholesterol, total cholesterol in HDL, HDL2 and medium HDL cholesterol, concentration of medium HDL cholesterol, cholesterol esters in medium HDL cholesterol, total lipids in medium HDL cholesterol and phospholipids in large and medium HDL cholesterol. These associations with miscarriage risk were all positive (adjusted RRs ranged between 1.36 and 1.44 per standard deviation increase). Mean diameter for LDL particles was inversely associated with risk of miscarriage (adjusted RR 0.71 per standard deviation increase). None of our findings for the metabolites reached our threshold for correction for multiple testing (p<0.003). There was some evidence of nonlinear relationship with three of the components (total lipids in small VLDL, cholesterol esters in very small VLDL, and phospholipids to total lipids ratio in IDL), with a p-value for the likelihood ratio test for nonlinearity ≤0.003. We therefore show the smoothed relationships with these three metabolites in *Extended data*, Appendix 4 ^14^.

The results did not notably change after adjustment for smoking or NEET status at age 18 in the subsample with this information available (approximately 80% of the total sample). ( *Extended data*, Appendices 5–7) ^14^. Only seven women were diagnosed with hypertension at 18 years of age, two women were diagnosed with high cholesterol, while none were diagnosed with diabetes. Excluding women who had hypertension or high cholesterol did not change the results. Finally, adjusting for the number of miscarriages reported by women’s mothers did not change the results ( *Extended data*, Appendices 8–10).

### Investigating the potential for selection bias

Restricting analyses to women who became pregnant by age 24 might introduce selection bias if there are unmeasured factors affecting both the likelihood of becoming pregnant and the likelihood of miscarriage, as depicted in *Extended data*, Appendix 11 ^14^. As demonstrated in the figure, selection bias would always induce an association between cardiometabolic health and miscarriage, that is in the same direction (i.e. positive or inverse) as the association between cardiometabolic health and becoming pregnant. However, in our study population, we found a positive association between BMI and the likelihood of becoming pregnant, and inverse associations of HDL-cholesterol, acetate, fatty acid levels and phospholipid levels with the likelihood of becoming pregnant ( *Extended data*, Appendices 12–14) ^14^. These are in the opposite direction to the associations we report between measures of cardiometabolic health (BMI, blood pressure, lipids, glucose and insulin) and the risk of miscarriage. Restricting the analysis to women who had become pregnant at an early age might therefore have biased our findings towards the null, but not away from the null.

Finally, we evaluated some known/established relationships in our study population to investigate the likelihood of selection bias. For example, the observed differences in cardiometabolic health and risk of miscarriage according to maternal education level in the study population were as expected, where women who had mothers with a higher educational level had lower mean BMI, lower blood pressure, and lower risk of miscarriage ( *Extended data*, Appendix 15) ^14^.

## Discussion

In this population-based study of young women who experienced a pregnancy between the ages of 18 and 24 years, we observed no strong evidence to suggest an effect of pre-pregnancy cardiometabolic health (BMI, blood pressure and metabolites) on risk of miscarriage.

A strength of our study is that we had measures on multiple aspects of cardiometabolic health before pregnancy. In particular, the inclusion of the metabolomic profile is unique. To our knowledge, there are only a small number of cohorts with similar data available, as most pregnancy cohorts recruit women who are already pregnant and therefore only have measurements from early pregnancy, which might not reflect pre-pregnancy levels. However, due to the small sample size and resulting uncertainty in effect estimates, our findings need to be replicated.

Both the measures of cardiometabolic health and the potential confounders (education, smoking, etc.) evaluated could have changed between when they were measured at 18 years of age and the time of conception. This could have introduced misclassification in the exposures and potential residual confounding. We did not have information on the gestational age or the birthweight of the fetus in cases of fetal death to verify and distinguish between what the women reported as a stillbirth as opposed to a miscarriage. We had to rely on the women´s report. It is therefore possible that there is a degree of misclassification between these two groups of fetal deaths.

Our sample consists of women who experienced a pregnancy at a relatively young age; mean age at first pregnancy in the UK is 30 ^18^. The proportion of women with a miscarriage in our study population (22%) is slightly higher than what has been reported for the general UK population (16%) ^19^. It is unlikely that this is driven by selection into the ALSPAC cohort, since participants have a higher socio-economic position, and are more likely to be Caucasian, compared to the general British population ^20^. Our study population (of women who became pregnant between the ages of 18 and 24) were substantially more likely to smoke (27% smoked daily) compared to women between 16 and 19 years of age in 2014 (16%) ^21^. This reflects the fact that women who become pregnant at a young age might also have a higher likelihood of various risk-seeking behaviors. Sampling variation is another potential explanation for the higher proportion of miscarriage in our study population. Our findings might therefore not be generalizable to all pregnant women. A second concern is that restricting our sample to pregnant women might result in selection bias as illustrated in *Extended data*, Appendix 11 ^14^. The prevalence of smoking at 18 years of age was higher among women who had experienced a pregnancy. There is some indication that the risk of miscarriage increases with smoking during pregnancy ^20^. However, through the use of directed acyclic graphs, we demonstrate that we are more likely to have underestimated than overestimated the associations of interest as a result of this restriction ( *Extended data*, Appendix 11) ^14^. Notably, we found that women who had experienced a pregnancy were of a similar socio-economic background to all female ALSPAC G1 participants ( *Extended data*, Appendix 3) ^14^. Lastly, the relationship between the cardiometabolic health measures, and the socio-economic differences in cardiometabolic health measures and risk of miscarriage were in line with the literature ^16, 17^.

Several studies indicate that women who are overweight or obese have an increased risk of miscarriage ^7, 8^. A study from the United Kingdom including 844 pregnancies from 491 women with a history of recurrent miscarriage was one of the first to report a nonlinear association between BMI and risk of miscarriage, with an almost fourfold increase in the risk among underweight women ^21^. Findings from the Danish National Birth Cohort (n= 23,821) subsequently provided evidence of an increased risk of miscarriage among underweight women, albeit of a more modest magnitude (OR 1.24) ^22^. We did not find strong evidence of a nonlinear association between BMI and miscarriage. This might reflect the age and/or BMI distributions in our study sample.

Findings from a preconception cohort revealed that women (mean age 29) with higher blood pressure before pregnancy may have an increased risk of pregnancy loss ^23^. In contrast, another study found evidence of lower blood pressure among women (mean age 35 years) with a history of recurrent miscarriages ^9^. In our study, we observed very weak evidence of an inverse association between SBP at age 18 and the risk of miscarriage, while the shape of the association with DBP was less clear.

Our study is the first to evaluate a broader metabolomics panel in relation to risk of miscarriage. We observed weak evidence of a positive association between total lipids and phospholipids in HLD-cholesterol with risk of miscarriage. A previous study of women with a history of recurrent miscarriages indicated greater levels of phosphatidylserine, and lower levels of phosphatidic acid, phosphatidylinositol and ganglioside mannoside 3 compared to women who had not experienced a miscarriage ^9^. Another study reported altered serum lipids profiles among women with a threatened miscarriage, including diminished concentrations of LDL cholesterol, total cholesterol, and phospoholipids ^24^. There are also a few previous studies which found that greater Apolipoprotein A-I might is associated with an increased likelihood of miscarriage ^25, 26^.

Potential explanations for an association between lower BMI and higher risk of miscarriage include nutrient deficiencies, including anaemia, folate, calcium, and iodine deficiencies, and altered placental development and structure ^27^. There is also evidence of metabolic regulators and reprogramming pathways of immune cell functions, which might contribute to a greater risk of infections and implantation failure among underweight women ^28^. Women undergo major hemodynamic changes during pregnancy, including increased blood volume, reduced cardiac output, lower heart rate, and decreased stroke volume ^29^. It might be that both lower and higher blood pressure before pregnancy result in maladaptation to hemodynamic changes, and increased risk of miscarriage.

Lipid metabolism may also play a role in ensuring a positive pregnancy outcome. It is clear that the metabolism of phospholipids changes during pregnancy ^30, 31^, but we do not fully understand if and what impact this may have on ensuring a healthy pregnancy. With regard to some of the other specific metabolites measured, Apolipoprotein A-I is an immunomodulator, and is shown to inhibit myeloid derived suppressor cell recruitment, which is known to regulate maternal pregnancy tolerance ensuring an intact pregnancy ^25, 26^.

Additional studies are necessary to understand whether the relationship between pre-pregnancy cardiometabolic health and risk of miscarriage differs by maternal age, to clarify whether there are certain age groups of women that could benefit from medical or lifestyle intervention targeting aspects of cardiometabolic health before they become pregnant to reduce the risk of miscarriage. Future studies could also look at the relationship between paternal cardiometabolic health and the risk of miscarriage as a negative control.

In conclusion, our findings indicate no strong evidence to support a relationship between pre-pregnancy cardiometabolic health and risk of miscarriage among young, healthy women who became pregnancy before age 24. Our findings need to be replicated in larger cohorts with pre-pregnancy measures of cardiometabolic health and in a wider age range.

## Data availability

ALSPAC data access is through a system of managed open access. The steps below highlight how to apply for access to the data included in this research article and all other ALSPAC data. The datasets presented in this article are linked to ALSPAC project number 2234, please quote this project number during your application. The ALSPAC variable codes highlighted in the dataset descriptions can be used to specify required variables.

- 1. Please read the ALSPAC access policy ( https://www.bristol.ac.uk/media-library/sites/alspac/documents/researchers/data-access/ALSPAC_Access_Policy.pdf) which describes the process of accessing the data and samples in detail, and outlines the costs associated with doing so.
- 2. You may also find it useful to browse our fully searchable research proposals database ( https://proposals.epi.bristol.ac.uk/), which lists all research projects that have been approved since April 2011.
- 3. Please submit your research proposal for consideration by the ALSPAC Executive Committee. You will receive a response within 10 working days to advise you whether your proposal has been approved.

If you have any questions about accessing data, please email alspac-data@bristol.ac.uk.

The ALSPAC data management plan describes in detail the policy regarding data sharing, which is through a system of managed open access. The study website also contains details of all the data that is available through a fully searchable data dictionary: http://www.bristol.ac.uk/alspac/researchers/data-access/data-dictionary/.

### Extended data

Figshare: 20200813 Supplement.pdf. https://doi.org/10.6084/m9.figshare.12800141 ^14^.

This file contains the following extended data:

- Appendix 1. Correlation between cardiometabolic health measures at 18 years of age among adult female offspring in the Avon Longitudinal Study of Parents and Children born between 1991–1992.
- Appendix 2. Illustration of study population among adult female offspring in the Avon Longitudinal Study of Parents and Children born between 1991–1992.
- Appendix 3. Distribution of female G1 participant’s background characteristics according to follow-up information available among adult female offspring in the Avon Longitudinal Study of Parents and Children born between 1991–1992.
- Appendix 4. Risk of miscarriage by metabolites for which there was some evidence of a nonlinear relationship among adult female offspring in the Avon Longitudinal Study of Parents and Children born between 1991–1992.
- Appendix 5. Association between body-mass index and blood pressure at age 18with risk of miscarriage by age 24 among individuals with information on educational/employment status at 18 years among adult female offspring in the Avon Longitudinal Study of Parents and Children born between 1991–1992.
- Appendix 6. Relative risks for miscarriage by 24 years of age according to components of lipoproteins among adult female offspring in the Avon Longitudinal Study of Parents and Children born between 1991–1992.
- Appendix 7. Relative risks for miscarriage by 24 years of age according to other components of the metabolomics panel among adult female offspring in the Avon Longitudinal Study of Parents and Children born between 1991–1992.
- Appendix 8. Directed acyclic graphs depicting four differences scenarios for how an unmeasured confounder might influence the association between cardiometabolic health and risk of miscarriage.
- Appendix 9. Association between body-mass index and blood pressure at age 18 with likelihood of becoming pregnant by 24 years of age among adult female offspring in the Avon Longitudinal Study of Parents and Children born between 1991̵1992.
- Appendix 10. Relative risks for becoming pregnant by 24 years of age according to components of lipoproteins among adult female offspring in the Avon Longitudinal Study of Parents and Children born between 1991–1992.
- Appendix 11. Relative risks for becoming pregnant by 24 years of age according to other components of the metabolomics panel among adult female offspring in the Avon Longitudinal Study of Parents and Children born between 1991–1992.
- Appendix 12. Association between maternal education qualifications with offspring body-mass index and blood pressure at age 18 and risk of miscarriage by age 24 among adult female offspring in the Avon Longitudinal Study of Parents and Children born between 1991–1992.

Data are available under the terms of the Creative Commons Zero "No rights reserved" data waiver (CC0 1.0 Public domain dedication).
