# Supplementary material for: Cardiometabolic health during early adulthood and risk of miscarriage: a prospective study
Source: Wellcome Open Res. 2021 Feb 12;5:205. Originally published 2020 Sep 3. [Version 2] doi: 10.12688/wellcomeopenres.16245.2 (PMC7888356; doi:10.12688/wellcomeopenres.16245.2)
Supplement: Peer review history

# Peer review: Reviewer response for version 2

**DOI:** 10.21956/wellcomeopenres.18312.r42608

**Published:** 2021-02-16

**Authors:** Maria Morales-Suarez-Varela

**Affiliations:** 1 Unit of Public Health and Environmental Care, Department of Preventive Medicine, University of Valencia, Valencia, Spain

After reviewing the article “Cardiometabolic health during early adulthood and risk of miscarriage: a prospective study”, compared to the previous article and the reviewers' response, I considered yes has made a substantial improvement in the presentation of the methodology and results of the study:

 The article does present better, clearer and more precise, already incorporated current literature.

 The design used is a novel and interesting design and it´s explained correctly.

 The methodology has been improved to make it more understandable, although I believe that the sample size of each group should be more clearly incorporated.

 Statistical analysis and lucky benefits correct.

 Taking into account the subject, it is very complex since it is multi-etiological (cardiometabolic health and the potential confounders (education, smoking, etc.) and the sample size as indicated by the reviewers is relatively small, despite which I consider that it can be useful of experience for the planning of future studies.

 Comments: I think that it is seen should be exposed more clearly about what is being worked and each moment of the study.

Is the work clearly and accurately presented and does it cite the current literature?

Partly

If applicable, is the statistical analysis and its interpretation appropriate?

Partly

Are all the source data underlying the results available to ensure full reproducibility?

Yes

Is the study design appropriate and is the work technically sound?

Yes

Are the conclusions drawn adequately supported by the results?

No

Are sufficient details of methods and analysis provided to allow replication by others?

Yes

Reviewer Expertise:

Epidemiology.

I confirm that I have read this submission and believe that I have an appropriate level of expertise to confirm that it is of an acceptable scientific standard.

# Peer review: Reviewer response for version 2

**DOI:** 10.21956/wellcomeopenres.18312.r42607

**Published:** 2021-02-15

**Authors:** Sohinee Bhattacharya

**Affiliations:** 1 Aberdeen Centre for Women's Health Research, University of Aberdeen, Aberdeen, UK

The authors have addressed issues raised in my previous report or explained why they cannot be addressed to my satisfaction. Just for clarification I had suggested that linkage with REDCAP would have given more valid data regarding pregnancy than self reports, particularly in relation to differentiation between miscarriage and termination of pregnancy.

Is the work clearly and accurately presented and does it cite the current literature?

Yes

If applicable, is the statistical analysis and its interpretation appropriate?

Yes

Are all the source data underlying the results available to ensure full reproducibility?

Partly

Is the study design appropriate and is the work technically sound?

Yes

Are the conclusions drawn adequately supported by the results?

Partly

Are sufficient details of methods and analysis provided to allow replication by others?

Yes

Reviewer Expertise:

Obstetric Epidemiology.

I confirm that I have read this submission and believe that I have an appropriate level of expertise to confirm that it is of an acceptable scientific standard.

# Peer review: Reviewer response for version 1

**DOI:** 10.21956/wellcomeopenres.17843.r41388

**Published:** 2020-11-25

**Authors:** Sohinee Bhattacharya

**Affiliations:** 1 Aberdeen Centre for Women's Health Research, University of Aberdeen, Aberdeen, UK

This interesting study utilising data collected from the G1 participants of the ALSPAC cohort found no association between cardiometabolic factors at age 18 and risk of miscarriage. The study design and analyses are appropriate taking account of the issue of multiple testing. However, the very small sample size and younger age of the participants mean that the generalizability of the findings is limited.

1. The authors report a higher risk of miscarriage in this cohort - 22% vs 15% in the general population despite the low risk age. Could this perhaps be explained by the higher prevalence of smoking, lower socioeconomic status and other characteristics that may have introduced a selection bias? I would have liked to see the BMI, blood pressure, smoking etc. compared between those with and without miscarriage in Table 1. 
2.  The authors state that pregnancy information was self reported and therefore subject to reporting bias. Why was pregnancy information not obtained by data linkage through REDCAP?
3. Are the results presented adjusted for all covariates including smoking (where 20% data were missing?
4. There is an association between maternal history of miscarriage and risk of miscarriage in their daughters (Woolner, 2020). As this cohort can possibly look at this association, it would have been good to present the interplay of genetic versus lifestyle factors in having a miscarriage.
5. My main concern is that the cohort is still too young to study this type of association as majority have not had a pregnancy yet and those who have are a very select group. Therefore the findings may change if the same analysis is repeated in the same cohort after another 5 years. I would urge the authors to wait till the cohort matures.

Is the work clearly and accurately presented and does it cite the current literature?

Yes

If applicable, is the statistical analysis and its interpretation appropriate?

Yes

Are all the source data underlying the results available to ensure full reproducibility?

Partly

Is the study design appropriate and is the work technically sound?

Yes

Are the conclusions drawn adequately supported by the results?

Partly

Are sufficient details of methods and analysis provided to allow replication by others?

Yes

Reviewer Expertise:

Obstetric Epidemiology.

I confirm that I have read this submission and believe that I have an appropriate level of expertise to confirm that it is of an acceptable scientific standard, however I have significant reservations, as outlined above.

# Response

**Published:** 2021-01-30

**Authors:** Maria C. Magnus

**Affiliations:** Norwegian Institute of Public Health, Norway

**Sohinee Bhattacharya**, Aberdeen Centre for Women's Health Research, University of Aberdeen, Aberdeen, UK 

 This interesting study utilising data collected from the G1 participants of the ALSPAC cohort found no association between cardiometabolic factors at age 18 and risk of miscarriage. The study design and analyses are appropriate taking account of the issue of multiple testing.

**Comment 1: However, the very small sample size and younger age of the participants mean that the generalizability of the findings is limited.**

 Response comment 1: We acknowledge the fact that the G1 participants in ALSPAC are relatively  young. This limitation is acknowledged in the discussion.

 Discussion (p. 12-13):

 “Our sample consists of women who experienced a pregnancy at a relatively young age; mean age at first pregnancy in the UK is 30.[6] Our findings might therefore not be generalizable to all pregnant women.  A second concern is that restricting our sample to pregnant women might result in selection bias as illustrated in Appendix 8. The prevalence of smoking at 18 years of age was higher among women who had experienced a pregnancy. There is some indication that the risk of miscarriage increases with smoking during pregnancy.[7] However, through the use of directed acyclic graphs, we demonstrate that we are more likely to have underestimated than overestimated the associations of interest as a result of this restriction (Appendix 8). Notably, we found that women who had experienced a pregnancy were of a similar socio-economic background to all female ALSPAC G1 participants (Appendix 3).  Lastly, the relationships between cardiometabolic health measures, and the socio-economic differences in cardiometabolic health measures and risk of miscarriage were in line with the literature.[8, 9] ”

**Comment 2: The authors report a higher risk of miscarriage in this cohort - 22% vs 15% in the general population despite the low risk age. Could this perhaps be explained by the higher prevalence of smoking, lower socioeconomic status and other characteristics that may have introduced a selection bias?**

 Response comment 2: As seen in many cohort studies, and in line with the population structure in the south west of the UK, participants in ALSPAC are actually of a higher socio-economic position and more likely to be Caucasian compared to the general British population. In response to this comment, we have added the following sentence into the discussion.

 Discussion (p. 12-13):

 “The proportion of women with a miscarriage in our study population (22%) is slightly higher than what has been reported for the general UK population (16%).[10] It is unlikely that this is driven by selection into the ALSPAC cohort, since participants have a higher socio-economic position, and are more likely to be Caucasian, compared to the general British population.[11] Our study population (of women who became pregnant between the ages of 18 and 24) were substantially more likely to smoke (27% smoked daily) compared to women between 16 and 19 years of age in 2014 (16%).[12] This reflects the fact that women who become pregnant at a young age might also have a higher likelihood of various risk-seeking behaviors. Sampling variation is another potential explanation for the higher proportion of miscarriage in our study population.”

**Comment 3: I would have liked to see the BMI, blood pressure, smoking etc. compared between those with and without miscarriage in Table 1.**

 Response comment 3: We have included a new table 1 illustrating the distribution of background characteristics and main cardiometabolic measures according to whether the woman experienced a miscarriage or not.

**Comment 4: The authors state that pregnancy information was self reported and therefore subject to reporting bias. Why was pregnancy information not obtained by data linkage through REDCAP?**

 Response comment 4: We apologise, but we are not sure that we follow this comment. Participants reported their obstetric history on xx questionnaires between the ages of 18 and 24 and at clinic assessments. RedCap is the data management system used in ALSPAC. We did not require linkage to external data sources such as medical records.

**Comment 5: Are the results presented adjusted for all covariates including smoking (where 20% data were missing?**

 Response comment 5: In the original draft, we did not show the results from the sensitivity analysis adjusting for the woman´s own smoking status. We now show the results from a combined sensitivity analysis both adjusting for own smoking status and NEET status at 18 years of age (Appendix 5-7). The results have been updated as follows.

 Results (p. 10):

 “The results did not notably change after adjustment for smoking or NEET status at age 18 in the subsample with this information available (approximately 80% of the total sample) (Appendix 5-7).”

**Comment 6: There is an association between maternal history of miscarriage and risk of miscarriage in their daughters (Woolner, 2020). As this cohort can possibly look at this association, it would have been good to present the interplay of genetic versus lifestyle factors in having a miscarriage.**

****

 Response comment 6: Thank you for raising this interesting issue. We have included an additional sensitivity analysis also adjusting for the mother´s report of miscarriages. This sensitivity analysis is now described in the methods and results.

 Methods (p. 7):

 “As there might be underlying genetic components influencing the risk of miscarriage, we also conducted a sensitivity analysis adjusting for maternal (G0) number of miscarriages.”

 Results (p. 10):

 “Finally, adjusting for the number of miscarriages reported by women’s mothers did not change the results (Appendix 8-10).”

**Comment 7:** **My main concern is that the cohort is still too young to study this type of association as majority have not had a pregnancy yet and those who have are a very select group. Therefore the findings may change if the same analysis is repeated in the same cohort after another 5 years. I would urge the authors to wait till the cohort matures.**

 Response comment 7: We understand the reviewer´s concern. This is why we conducted an extensive evaluation of the potential influence of a selection bias. We agree that the research question should be re-visited after the cohort has matured a bit more. However, this will take time, and we think that there is value to presenting the results as we see them so far. In response to this comment, we have tempered our conclusion as follows.

 Abstract (p. 2):

 “Our findings indicate no strong evidence to support a relationship between pre-pregnancy cardiometabolic health and risk of miscarriage in young, healthy women who became pregnant before age 24. Future studies are necessary that are able to evaluate this question among women who have a wider age range.”

 Discussion (p. 15):

 “In conclusion, our findings indicate no strong evidence to support a relationship between pre-pregnancy cardiometabolic health and risk of miscarriage among young, healthy women who became pregnancy before age 24. Our findings need to be replicated in larger cohorts with pre-pregnancy measures of cardiometabolic health and in a wider age range.”

# Peer review: Reviewer response for version 1

**DOI:** 10.21956/wellcomeopenres.17843.r40288

**Published:** 2020-09-09

**Authors:** Maria Morales-Suarez-Varela

**Affiliations:** 1 Unit of Public Health and Environmental Care, Department of Preventive Medicine, University of Valencia, Valencia, Spain

Thank you very much for allowing me to review the research article entitled “Cardiometabolic health during early adulthood and risk of miscarriage: a prospective study”.

 The objective of this work was to investigate the risk of miscarriage according to pre-pregnancy cardiometabolic health. Concretely to examine whether pre-pregnancy BMI, blood pressure, fasting insulin, and metabolomic profile at age 18 are associated with miscarriage risk in young, healthy women who became pregnant before 24 years of age.

 Methodologically the study is carried out on participants in the Avon Longitudinal Study of Parents and Children (ALSPAC) (UK).

 Concluding that pre-pregnancy cardiometabolic health in late adolescence was not associated with miscarriage risk in young, healthy women who became pregnant before age 24.

**Comments:**

 It is a study with a very important objective today, as we see an increase in early pregnancies.

**Introduction:**

 It should be more extensive, especially the concept to be studied "risk of miscarriage" and the current knowledge on this topic in relation to the cardiometabolic factors studied should be defined.

**Methodology and results:**

 A figure with a diagram of the study participants would facilitate understanding (G1 and G2).

 Defining the studied concept "risk of miscarriage" may or may not be an abortion, and if it can be included at what time of pregnancy, first trimester, Second ...

 The measurements used are at 18 years of age, but these do not have to be the same pre-pregnancy, these two moments should be evaluated in the parameters studied. The same happens with the educational level, the socioeconomic level, and the smoking habit.

 A table comparing the characteristics of women with "risk of miscarriage" and women who successfully terminated their pregnancy would be essential to understand the study. In the statistical analysis, it should be more clearly indicated when crude RR is used and when adjusted and why covariates.

 In the results, indicate the statistically significant results. Adjusted RR would be convenient, since "risk of miscarriage" is multifactorial.

**Discussion:**

 The authors are aware of the strengths and weaknesses of the study.

**Overall assessment:**

 The study raises a very interesting objective and uses a prominent project database. The design should assess a comparison between the situation of pregnant women at 18 years of age and their situation prior to pregnancy. However, the available sample size is small for the proposed objective, so its conclusions are risky.

Is the work clearly and accurately presented and does it cite the current literature?

Partly

If applicable, is the statistical analysis and its interpretation appropriate?

Partly

Are all the source data underlying the results available to ensure full reproducibility?

Yes

Is the study design appropriate and is the work technically sound?

Yes

Are the conclusions drawn adequately supported by the results?

No

Are sufficient details of methods and analysis provided to allow replication by others?

Yes

Reviewer Expertise:

Epidemiology

I confirm that I have read this submission and believe that I have an appropriate level of expertise to confirm that it is of an acceptable scientific standard, however I have significant reservations, as outlined above.

# Response

**Published:** 2021-01-30

**Authors:** Maria C. Magnus

**Affiliations:** Norwegian Institute of Public Health, Norway

**Maria Morales-Suarez-Varela**, Unit of Public Health and Environmental Care, Department of Preventive Medicine, University of Valencia, Valencia, Spain 

 Thank you very much for allowing me to review the research article entitled “Cardiometabolic health during early adulthood and risk of miscarriage: a prospective study”.

 The objective of this work was to investigate the risk of miscarriage according to pre-pregnancy cardiometabolic health. Concretely to examine whether pre-pregnancy BMI, blood pressure, fasting insulin, and metabolomic profile at age 18 are associated with miscarriage risk in young, healthy women who became pregnant before 24 years of age.

 Methodologically the study is carried out on participants in the Avon Longitudinal Study of Parents and Children (ALSPAC) (UK).

 Concluding that pre-pregnancy cardiometabolic health in late adolescence was not associated with miscarriage risk in young, healthy women who became pregnant before age 24.

**Comments:**

 It is a study with a very important objective today, as we see an increase in early pregnancies.

**Comment 1:**

**Introduction**

**It should be more extensive, especially the concept to be studied "risk of miscarriage" and the current knowledge on this topic in relation to the cardiometabolic factors studied should be defined.**

 Response comment 1: We have expanded the introduction as requested.

 Introduction (p. 3):

 “Several studies have found that women who are overweight or obese have an increased risk of miscarriage, while others report a potential nonlinear relationship with an increased risk in underweight women as well.[1, 2]  There is conflicting evidence of a relationship between blood pressure and risk of miscarriage, with one preconception cohort indicating a positive association with pregnancy loss, [3] while another study reported a lower blood pressure among women with a history of recurrent miscarriage.[4] The evidence of a relationship between lipid levels and risk of miscarriage is scarce. Animal studies support the notion that treatment of high cholesterol with statins may reduce the risk of miscarriage.[5]”

**Methodology and results**

**Comment 2: A figure with a diagram of the study participants would facilitate understanding (G1 and G2).**

 Response comment 2: We have created a figure illustrating the design of the study and the generations participating in ALSPAC (see Figure 1).

**Comment 3: Defining the studied concept "risk of miscarriage" may or may not be an abortion, and if it can be included at what time of pregnancy, first trimester, Second...**

 Response comment 3: We have incorporated the following limitation in the discussion.

 Methods (p. 6):

 “The outcome of any pregnancies were classified as “baby born alive”, “baby born stillborn”, “termination of an unwanted pregnancy”, “termination for medical reasons” and “miscarriage”. No additional information on the gestational week when the pregnancy ended or the birthweight of the offspring was available to better distinguish between stillbirth and miscarriage.”

 Discussion (p. 12):

 “We did not have information on the gestational age or the birthweight of the fetus in cases of fetal death to verify and distinguish between what the women reported as a stillbirth as opposed to a miscarriage. We had to rely on the women´s report. It is therefore possible that there is a degree of misclassification between these two groups of fetal deaths.”

**Comment 4: The measurements used are at 18 years of age, but these do not have to be the same pre-pregnancy, these two moments should be evaluated in the parameters studied. The same happens with the educational level, the socioeconomic level, and the smoking habit.**

 Response comment 4: Thank you for raising these important points. We agree that both the measures of pre-pregnancy cardiometabolic health and the potential confounders evaluated may have changed between the time of measurement and conception. This has now been acknowledged as a limitation.

 Discussion (p. 12):

 “Both the measures of cardiometabolic health and the potential confounders (education, smoking, etc.) evaluated could have changes between when they were measured at 18 years of age and the time of conception. This could have introduced misclassification in the exposures and potential residual confounding.”

**Comment 5: A table comparing the characteristics of women with "risk of miscarriage" and women who successfully terminated their pregnancy would be essential to understand the study. In the statistical analysis, it should be more clearly indicated when crude RR is used and when adjusted and why covariates.**

 Response comment 5: In response to this comment, we have created a new Table 1 which illustrates the distribution of background characteristics among women who did and did not experience a miscarriage.

 Results (p. 9):

 “The distribution of background characteristics according to whether the woman experience a miscarriage or not is shown in Table 1.”

**Comment 6: In the results, indicate the statistically significant results. Adjusted RR would be convenient, since "risk of miscarriage" is multifactorial.**

 Response comment 6: All results presented are adjusted. In response to this comment, we have carefully gone through the results to clarify this.

**Discussion:**

**Comment 7: The authors are aware of the strengths and weaknesses of the study.**

 Response comment 7: We have elaborated a bit more on the weaknesses of the study in response to your comments and those of the other reviewer (see p. 12-13).

**Overall assessment:**

**Comment 8: The study raises a very interesting objective and uses a prominent project database. The design should assess a comparison between the situation of pregnant women at 18 years of age and their situation prior to pregnancy. However, the available sample size is small for the proposed objective, so its conclusions are risky.**

 Response comment 8: We acknowledge the fact that ALSPAC G1 offspring (those born in 1991 and 1992) are still relatively young, and as a result only a small proportion of them have started having children of their own. In response to this comment and those of the other reviewer, we have tempered our conclusion as follows.

 Abstract (p. 2):

 “Our findings indicate no strong evidence to support a relationship between pre-pregnancy cardiometabolic health and risk of miscarriage in young, healthy women who became pregnant before age 24. Future studies are necessary that are able to evaluate this question in samples with a wider age range.”

 Discussion (p. 15):

 “In conclusion, our findings indicate no strong evidence to support a relationship between pre-pregnancy cardiometabolic health and risk of miscarriage among young, healthy women who became pregnancy before age 24. Our findings need to be replicated in larger cohorts with pre-pregnancy measures of cardiometabolic health and in a wider age range.”
